# Attenuated Boundary Extension Produces a Paradoxical Memory Advantage in Amnesic Patients

**DOI:** 10.1016/j.cub.2012.01.001

**Published:** 2012-02-21

**Authors:** Sinéad L. Mullally, Helene Intraub, Eleanor A. Maguire

**Affiliations:** 1 Wellcome Trust Centre for Neuroimaging, Institute of Neurology, University College London, 12 Queen Square, London WC1N 3BG, UK; 2 Department of Psychology, University of Delaware, 108 Wolf Hall, Newark, DE 19716, USA

## Abstract

**Background:**

When we view a scene, we construct an internal representation of the scene that extends beyond its given borders. This cognitive phenomenon is revealed by a subsequent memory error when we confidently misremember the extended scene instead of the original. This effect is known as “boundary extension” and is apparent in adults, children, and babies.

**Results:**

Here we show that seven patients with selective bilateral hippocampal damage and amnesia, who cannot imagine spatially coherent scenes, displayed attenuated levels of boundary extension on three separate measures. Paradoxically, this reduced boundary extension resulted in better memory for the stimuli compared with matched control participants, because the patients' recall was less encumbered by the boundary extension error. A further test revealed that although patients could generate appropriate semantic, conceptual, and contextual information about what might be beyond the view in a scene, their representation of the specifically spatial aspect of extended scenes was markedly impoverished.

**Conclusions:**

The patients' superior memory performance betrayed a fundamental deficit in scene processing. Our findings indicate that the hippocampus supports the internal representation of scenes and extended scenes when they are not physically in view, and this may involve providing a spatial framework in scenes. We suggest that interference with the ability to internally represent space may prevent the construction of spatially coherent scenes, with possible consequences for navigation, recollection of the past, and imagination of the future, which depend on this function.

## Highlights

► Patients with hippocampal damage and amnesia cannot imagine spatially coherent scenes ► Boundary extension (BE) is when people remember seeing more of a scene than was shown ► Amnesic patients had attenuated BE, which paradoxically permitted better memory ► Hippocampus supports scene representations, possibly supplying the spatial framework

## Introduction

Decades of animal work have shown that a brain structure called the hippocampus plays an essential role in constructing internal spatial representations of the environment [1–3]. In humans, exactly how this spatial function relates to the acknowledged importance of the hippocampus for recalling past experiences [4] remains a key unanswered question in neuroscience [2, 5, 6]. In recent years, the importance of the hippocampus has been amplified further with the realization that it is not only necessary for recalling the past, but also enables imagination of fictitious and future scenes and events [7–9]. Hassabis et al. [10] found that patients with bilateral hippocampal damage and amnesia could not imagine either fictitious or future scenes (see also [11–14]); their constructions were fragmented and lacked spatial coherence. This led to the proposal that the hippocampus may facilitate the construction of complex spatial contexts or scenes into which event details are bound, and this scene construction process may underpin functions such as navigation, recalling the past, and imagining the future [6].

**Figure 1:**
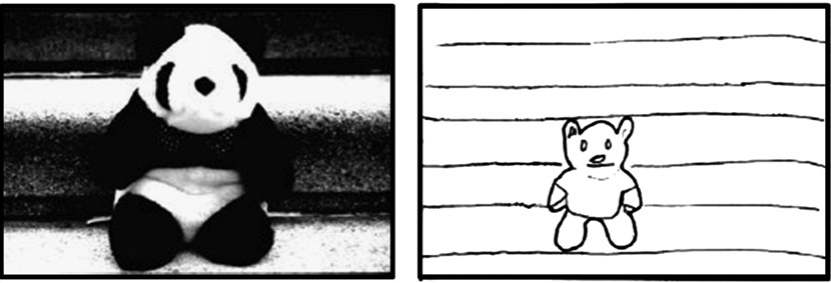
A Demonstration of Boundary Extension The left panel shows the studied photograph (i.e., a close-up view of a scene) and the right panel shows the scene as subsequently drawn from memory moments later by a control participant. The drawing clearly depicts a more extended expanse of background than was evident in the original stimulus (taken from [24]).

Here we sought to test the proposal that the hippocampus is critical for scene construction in a novel way. When we view a scene, we construct an internal representation of the scene that extends beyond its given borders. This powerful cognitive phenomenon is revealed in a memory error known as “boundary extension” (BE; [15]). Figure 1 illustrates this effect, where a healthy participant, having studied a picture of a simple scene, drew the scene from memory moments later and included much more surrounding background than was present in the studied stimulus. Interestingly for our purpose, BE occurs specifically in relation to scenes, but not for single acontextual objects [16, 17].

Boundary extension is a robust effect apparent in all populations sampled—adults [15, 18], children [18, 19], and even babies [20]. It involves a two-stage process. The first stage is constructive in nature and occurs because when we initially encounter a scene, we are not limited to the information that is in front of our eyes, but have access to an automatically constructed and implicitly maintained internal representation of the scene. This latter representation extends well beyond the borders of the given scene and provides an overarching framework into which we rapidly embed what is currently in our field of view [21]. This is a highly adaptive process that supports our experience of a continuous and coherent world, despite it being amassed from discontinuous sensory input. That this initial construction stage has occurred is revealed by a subsequent memory error at recall. Here, in the absence of the studied view, we mistakenly attribute the anticipated information in the extended scene representation as having been seen before. The fact that the studied view need only be absent for as little as 42 ms for BE to be apparent [22, 23] underscores the online and spontaneous nature of this effect.

Because BE captures something automatic and fundamental about our interaction with scenes, and necessarily depends on intact scene construction ability, it is of interest to determine whether patients with bilateral hippocampal damage and amnesia show normal BE. If they exhibit BE similar to control participants, it would suggest that they have access to coherent scene representations beyond the studied view. If, however, they show reduced or absent BE, this may indicate a fundamental problem generating internalized scene representations. Critically, the latter finding of attenuated BE could not be attributed to a failure of memory between study and test as, paradoxically, amnesic patients would perform more accurately at recall than control participants, because their poor scene construction beyond the edges of the given view would actually serve to minimize BE. Thus, BE afforded us a unique opportunity to investigate scene processing against the background of profound memory impairment, potentially offering additional leverage on the question of what function the hippocampus performs.

We employed three classic tests of boundary extension. The first was a modified version of a rapid serial visual presentation (RSVP) task [23, 24], the second a drawing task [15, 25], and the third was a haptic (tactile) BE paradigm [26]. The latter enabled us to assess whether the same pattern of results would be observed in another modality in the absence of visual input. Given that BE is evident after even the briefest intervals [22, 23], it was possible to adapt all three tasks for use in memory-impaired individuals, such that recall was always assessed immediately after the presentation of each scene stimulus, thus eschewing a requirement for long-term memory. We also devised a further “scene probe” test. This was important because it allowed us to explore in detail the nature of participants' internal representations of what might be beyond the current view for a given scene specifically in terms of conceptual, contextual, and spatial sources of information.

In order to functionally localize any effects to the hippocampus specifically, it is necessary to assess patients with damage restricted to the two hippocampi alone (as far as this is possible to establish with current techniques). We tested seven such patients whose selective, bilateral hippocampal lesions were confirmed using high-resolution structural magnetic resonance imaging (MRI) coupled with two independent measurement techniques (see Experimental Procedures; see also Supplemental Experimental Procedures and Figure S1 available online). These predominantly young, high-functioning patients had a selective and severe episodic memory impairment (see Table S1) and a significant deficit in the ability to imagine spatially coherent scenes. The question was, would they show normal boundary extension?

## Results

### Scene Construction

We first assessed whether this group of patients would show scene construction deficits similar to patients reported previously. Using an established paradigm [10], participants were required to construct newly imagined fictitious scenes in response to a verbal cue. The experiential index (a measure of the richness of the imagined scenes) was significantly lower in the patients (24.22 ± 17.84) relative to controls (47.52 ± 7.08; *U* = 6.0, *Z* = −3.04, p = 0.002) (Figure S2A). Similarly, patients' scenes were significantly more fragmented and less spatially coherent (−1.61 ± 4.09) than those imagined by controls (3.58 ± 2.04; *U* = 13.5, *Z* = −2.41, p = 0.016) (Figure S2B). Therefore, just as with previous amnesic patients (e.g., [10]), these new patients were also unable to imagine spatially coherent scenes.

### Boundary Extension

Having established that our patients had severe problems imagining spatially coherent scenes, we next tested boundary extension in three different ways. All participants were naive to the concept of BE.

#### Rapid Serial Visual Presentation Task

**Figure 2:**
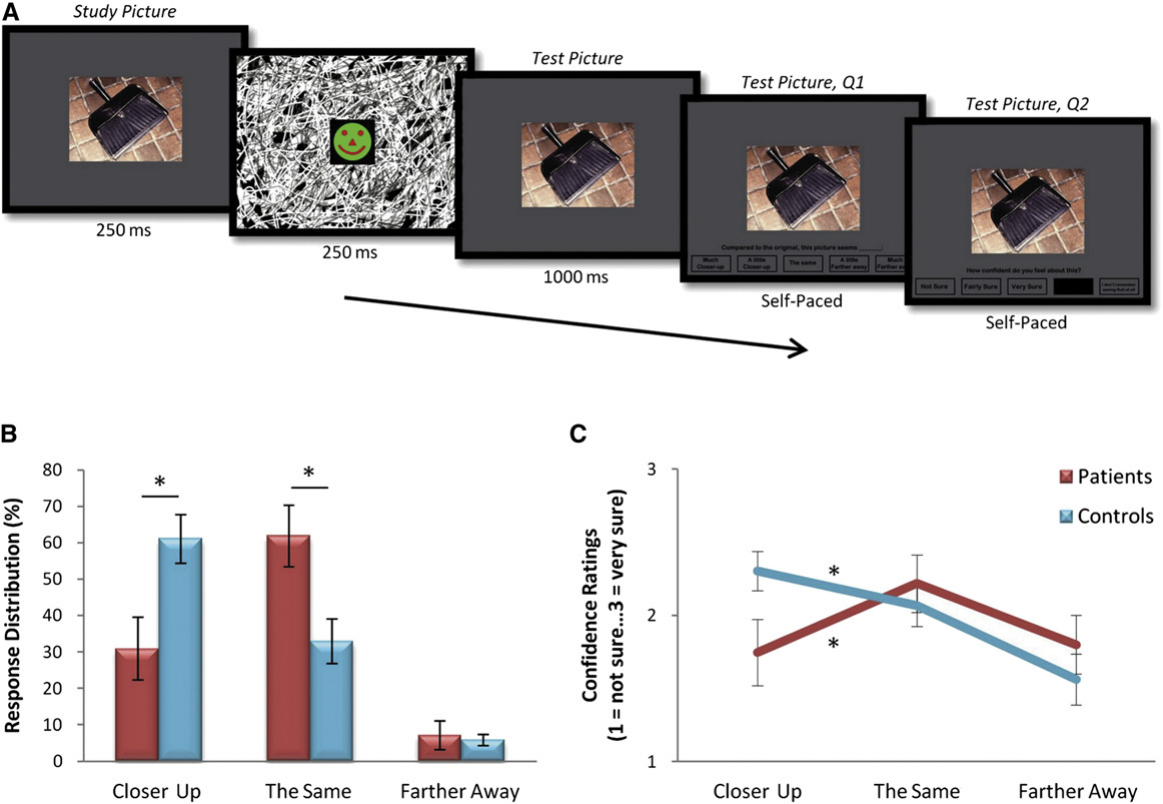
Rapid Serial Visual Presentation BE Task (A) Timeline of an example trial. The initial picture (i.e., a close-up photograph of a scene) comprised a single, nonoccluded, centrally positioned object and was presented on the computer screen for 250 ms, followed by a briefly presented (250 ms) dynamically changing mask [23]. The second picture (which was always identical to the original picture) immediately followed the mask. The task was to rate the second picture relative to the first. There were five options, i.e., “much closer up,” “a little closer up,” “the same” (the correct answer), “a little farther away,” or “much farther away,” and participants completed 24 trials. (B) The proportion of trials classified as either “closer up,” “the same” (correct answer), or “farther away” was calculated and represented as a percentage response distribution score (percentage of responses made in each category relative to the total number of responses made). BE is revealed by disproportionally large number of incorrect “closer-up” responses. Overall, control participants made significantly more erroneous BE (i.e., “closer-up”) responses, whereas the patients made significantly more accurate (i.e., “the same”) responses. (C) Participants also reported how confident they were about their decision using a three-point scale (1 = “not sure,” 2 = “fairly sure,” 3 = “very sure”) and mean confidence ratings were calculated for each of the three response types. Control participants were significantly more confident when making erroneous “closer-up” responses, and patients were significantly more confident about their correct “the same” response. An “I don't remember seeing that at all” option was also included but never selected. Data are presented as means ± 1 SEM; ^∗^p < 0.05. See also Figure S3.

Participants were presented with two scene pictures in rapid succession separated by a briefly visible visual noise mask (initial scene presentation = 250 ms; masked interstimulus interval = 250 ms; Figure 2A) and were simply asked to rate whether the second picture depicted a closer-up, the same, or a farther-away view of the scene than was shown in the original picture. Unbeknownst to participants, the two pictures were always identical, and thus, all picture pairs should have been rated as the same. However, on a high proportion of trials in this type of task, healthy participants often rate the second picture as closer-up than the first picture, thus exhibiting BE [24]. This is because when they initially view a scene, participants typically imagine the extended environment surrounding the scene [21]. When this more expansive representation is subsequently compared with the second “test” picture, although it is identical to the initial picture viewed only 250 ms previously, the second picture is consistently believed to depict a closer-up scene.

As anticipated, our control participants classified the majority of trials as closer-up (61.1% ± 23.05%). By contrast, the patients classified less than a third of trials as closer-up (30.95% ± 22.8%) and significantly fewer than the controls (*U* = 15.5, Z = −2.26, p = 0.024). In addition, patients correctly identified 61.9% (±22.36%) of trials as the same in comparison to only 32.99% (±21.2%) of trials classified as such by controls (Figure 2B, see also Figure S3). Again, this difference was significant (*U* = 14.5, Z = −2.33, p = 0.02). Notably, the patients' errors, when they made them, were not random, but were in the same direction as the controls; i.e., they rated pictures as closer up. Farther away classifications accounted for only a small number of responses (patients: 7.14% ± 10.4%; controls 5.9% ± 5.17%) and did not differ between groups (*U* = 38.5, Z = −0.31, p = 0.76). Thus, the patients' attenuated BE was not simply because they failed to recall the studied picture at test, because this would have led to an equal distribution of responses across the three categories, which was clearly not the case. Therefore, although both groups made BE errors, controls made significantly more BE errors than patients, presumably because when viewing a study picture, their internal representation of the studied scene went well beyond what was in front of them. By contrast, patients' performance was significantly more accurate because their impaired scene construction ability presumably diminished their capacity to construct the extended scene representations, rendering their performance on this task less susceptible to the BE error.

Participants also rated how confident they felt about each decision in the RSVP task. Patients were significantly more confident on trials they correctly identified as the same (2.22 ± 0.52; 1, not sure…3, very sure) relative to the trials they erroneously identified as closer up (1.75 ± 0.6; Z = −2.20, p = 0.028), whereas controls were significantly more confident about their closer-up choices (2.3 ± 0.46) than their “the same” judgments (2.07 ± 0.46; Z = −2.19, p = 0.028). Thus, the groups' confidence ratings mirrored the patterns observed in their task performance, with the patients being more confident about their correct responses, whereas the controls were more confident about their erroneous (BE-influenced) responses (Figure 2C).

#### Drawing Task

**Figure 3:**
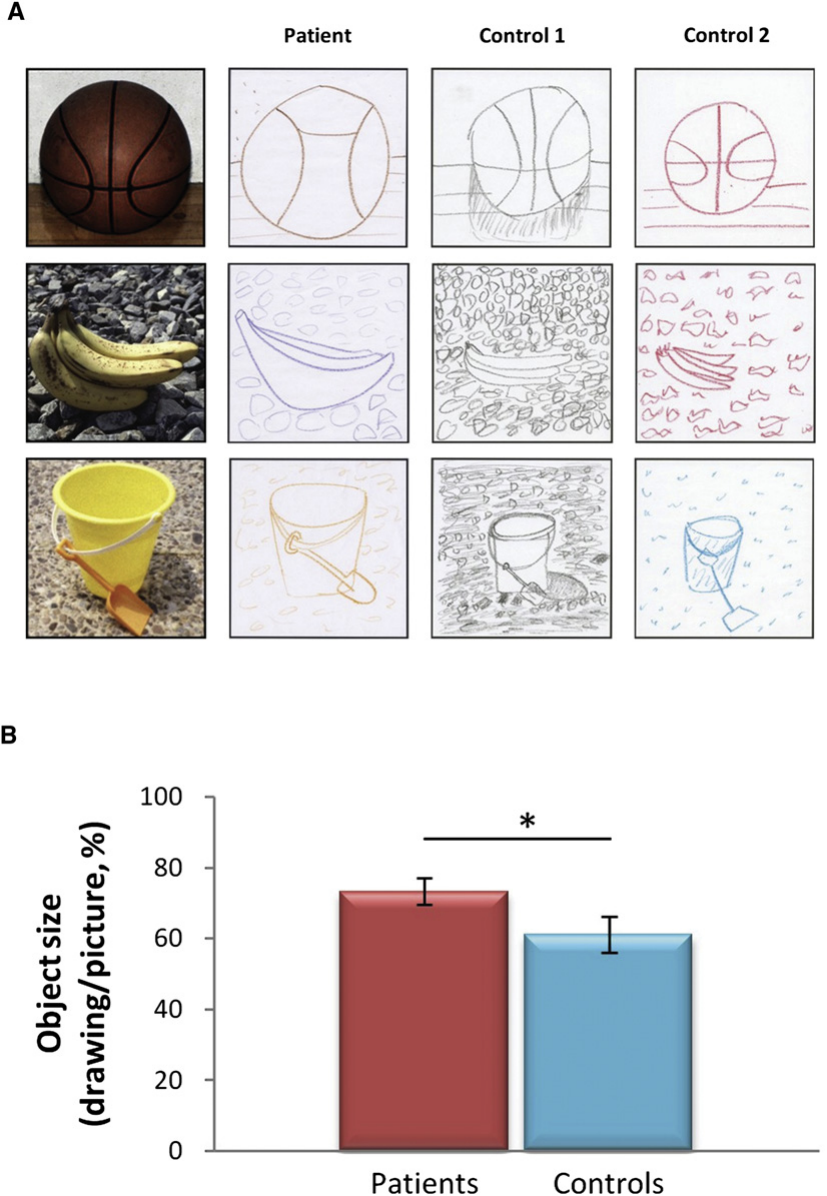
BE Drawing Task (A) The left panel displays the three scene stimuli. Each scene photograph was surrounded by a 6 × 6 inch black border and was studied for 15 s and immediately drawn from memory in a 6 × 6 inch response square. Example drawings by a patient and her two matched control participants are displayed in the middle and left panels. In both control participants' drawings, more background is clearly depicted than was present in the original stimuli. This represents greater BE and was quantified in terms of a percentage area decrease in object size (calculated by tracing along the outer borders of the objects using Adobe Photoshop C54 and measuring the area in pixels) in the remembered relative to the original object size. (B) Overall, patients showed significantly less boundary extension than control participants. Data are presented as means ± 1 SEM; ^∗^p < 0.05. See also Figure S4.

Having observed significantly attenuated BE in the RSVP task, we then employed a completely different test to examine the robustness of this finding. Participants studied photographs of scenes, one at a time for 15 s, and then immediately drew a scene from memory (Figure 3A). By examining the proportional size of the drawn object relative to the original, we obtained a quantitative measure of BE and an insight into the extended scene representations driving the BE error [15, 25, 27]. The control participants, as expected, included more of the scene than was actually present in the original stimuli, thus reducing the area covered by the object so that it was only 61.16% of its original size (±17.75%; t = −7.58, df = 11, p = 0.001; Figure 3A). The patients also included more of the scene than was shown, reducing the area covered by the object so that it was 73.42% of its original size (±10.03%; t = −7.01, df = 6, p = 0.001; Figure 3A), but this was significantly less of a reduction compared to the controls (*U* = 17, Z = −2.11, p = 0.035, Figure 3B). Thus, the patients' recall, despite their significant memory problems, was not as vulnerable to distortion by the BE error, resulting in the production of more proportionally veridical drawings than those constructed by the controls.

Of note, a group of independent assessors blindly rated the drawings, deciding whether each drawing was made by a healthy control participant or a person with a memory problem. The patients and controls could not be differentiated in terms of the overall quality of their drawings (see Supplemental Experimental Procedures, and Figure S4). Another group of independent assessors blindly rated the specific quality and detail of the separate elements of the drawings (i.e., the objects and the backgrounds). Again, there was no significant difference in the quality and detail of either the objects or the backgrounds for patients and controls (see Supplemental Experimental Procedures).

#### Haptic Task

**Figure 4:**
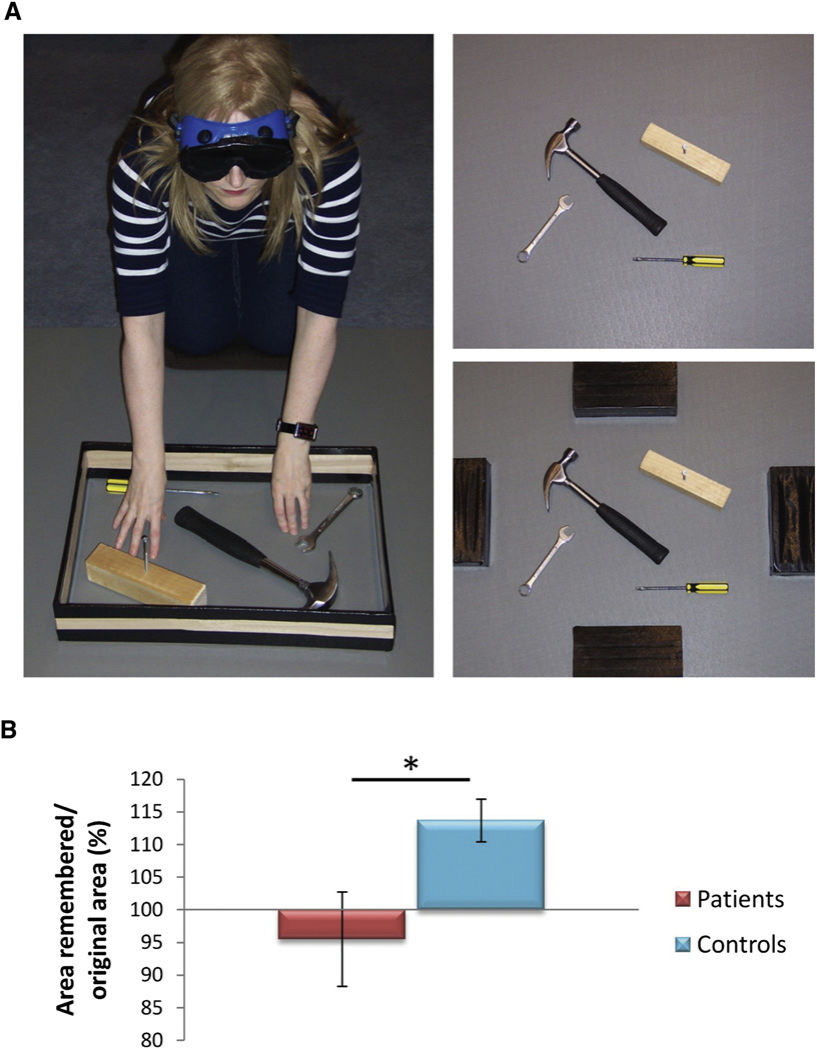
Haptic BE Task (A) Participants explored three distinct scenes, each presented within a wooden border (left panel), for 30 s using touch alone. The border was then removed (upper right panel) and participants (still blindfolded) were asked to indicate the original location of each border using large markers (right lower panel). (B) BE was defined in terms of an increase in the reconstructed scene area relative to the original scene's size. Compared to the control group, patients showed significantly less boundary extension. Data are presented as means ± 1 SEM; ^∗^p < 0.05.

The results of the two BE tasks above revealed a reliable attenuation of the BE error in amnesic patients relative to control participants. We then wondered whether these between-group differences were restricted to the visual domain. In order to test this, we utilized a haptic BE paradigm, which required participants to study and recall the dimensions of studied scenes using touch alone (Figure 4A). A BE error results in the misplacement of the borders so that the scene area at recall is significantly larger than the original scene area, as indeed was evident in the control group (mean 113.03% ± 11.6%; t = 3.89, df = 11, p = 0.003, [Figure 4B]). Critically, the patients failed to show BE (mean 95.5% ± 19.12%; t = −0.62, df = 6, p = 0.56), and their performance differed significantly from that of the controls (*U* = 17, Z = −2.11, p = 0.035). The patients' lack of BE in the haptic domain (i.e., in the absence of visual input) argues against specific visuoperceptual scene deficits [28] or eye movement differences driving the disparity between the groups.

### Scene Probe Task

**Figure 5:**
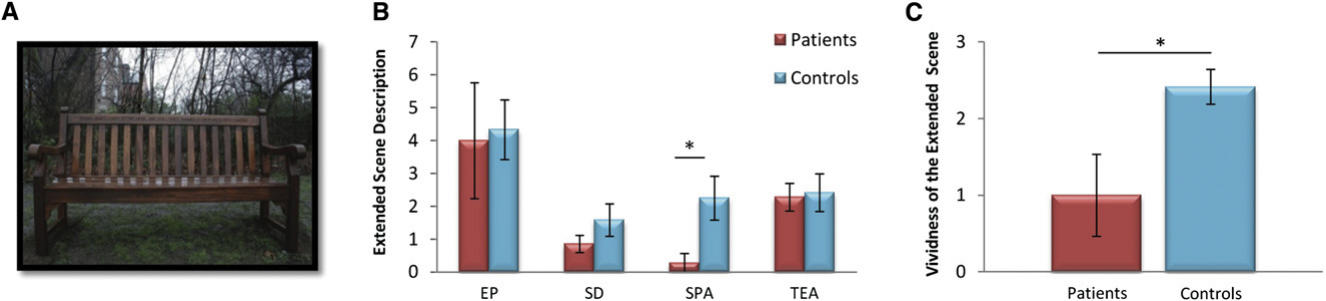
Scene Probe Task (A) Depicts the scene stimulus used. Participants were asked to describe out loud a number of components of this scene, including the object and background, and to name the type of place where the photograph was taken. (B) They were also asked to describe what the scene might be like beyond the boundaries of the current view. Verbal descriptions were recorded and later transcribed. Information content was classified into four categories according to an established protocol [10], namely entities present (EP), sensory descriptions (SD), spatial references (SPA), and thoughts/emotions/actions (TEA). Relative to controls, patients produced significantly fewer spatial references in these descriptions. There were no differences in the production of the other detail types. (C) Participants were also asked whether they were actually able to visualize the extended scene in their imagination and to rate its vividness using a 3-point scale (1 = low vividness, 2 = medium vividness, 3 = high vividness). If they were unable to visualize anything, they were given a score of 0. Compared with the control participants, patients reported their imagined extended scenes were significantly less vivid. Data are presented as means ± 1 SEM; ^∗^p < 0.05. See also Figure S5.

The scene construction and boundary extension results show that the patients had a significant difficulty with generating representations of scenes and extended scenes. Scenes are complex and comprise multiple elements, and so using a scene probe task we also attempted to ascertain what aspect of scenes might be particularly compromised in these patients with bilateral hippocampal damage. A close-up photograph of a scene was presented to participants on a computer screen (Figure 5A). Participants were asked to describe out loud a number of components of the scene. All patients were able to provide confident and accurate descriptions of the scene stimulus, in line with those given by controls (see an example response in Figure S5). This confirmed that the patients had no difficulty in perceiving pictures of scenes or providing rich narratives [13]. In response to the question “What sort of a place do you think this picture was taken in?” the patients were able to locate the scene within an appropriate context (Patient A: “an urban surrounding,” Patient B: “a park,” Patient C: “a cemetery or a park,” Patient D: “a park or a village green,” Patient E: “a cemetery or church grounds,” Patient F: “a square or a local park in the middle of a city,” Patient G: “a small park in an urban setting”). This argues against the possibility that the patients were unable to bring to mind relevant contextual associations. Similarly, in response to the request to imagine taking a few steps back from the camera's current position and describe the scene beyond the current view, patients were able to list numerous contextually relevant items, associate them with each other, and associate them with the context. In so doing, the patients showed that they were clearly able to appropriately attend to and anticipate what might be beyond the view in the scene.

When their descriptions of what might be beyond the view were formally assessed using an established protocol [10], we observed no difference in the number of entities proposed to be present beyond the view (*U* = 35, *Z* = −0.60, p = 0.55), the number of sensory descriptions (*U* = 32, *Z* = −0.88, p = 0.38), or thoughts, actions, or emotions (*U* = 38, *Z* = −0.35, p = 0.73) recounted by the patients relative to the control participants (Figure 5B). However, in stark contrast to controls, patients omitted spatial references almost entirely from their descriptions of what was likely to be beyond the view (patients 0.43 ± 0.79; controls 2.25 ± 2.3; *U* = 18.5, *Z* = −2.08, p = 0.038). Patients also rated the vividness of these extended imagined scenes as significantly lower than controls (patients 1.0 ± 1.41; controls 2.42 ± 0.79; *U* = 18, *Z* = −2.16, p = 0.03; Figure 5C). In fact, most patients reported not being able to visualize anything at all (see example in Figure S5). Thus, although patients could provide rich semantic, associative, and contextual information, they specifically could not imagine the spatial structure of the scene, echoing their performance on the scene construction task. They could not truly visualize what might be beyond the current view.

## Discussion

In this study we availed ourselves of a unique cognitive phenomenon, boundary extension [15, 21, 29], whereby healthy people consistently remember a greater expanse of a scene than was shown in the given view. In contrast to matched controls, patients with apparently selective bilateral hippocampal damage and amnesia showed a striking attenuation of BE across three different tasks. Paradoxically, reduced BE meant that the amnesic patients exhibited superior memory (an exceptional occurrence; see [30–34], and also [35, 36] for recent reviews) for the extent of the background context present in the stimuli. This result enabled us to identify a specific deficit in scene processing that cannot be attributed to a simple failure of memory between study and test, suggesting that the hippocampus supports the internal representation of scenes (scene construction) and extended scenes (boundary extension) when they are not physically in view.

Because there are specific situations where attention can attenuate BE [37, 38], we considered a number of alternative explanations for our findings. Might it be that, given their difficulties with spatial coherence in scenes, the patients attended to the objects at the expense of the background? If this was the case, then we would have expected boundary errors to be random (i.e., equal number of closer-up and farther away responses in the RSVP task), and poorer recall of background content. However, this is not what we found. Patients' errors, where they made them, were unidirectional and nonrandom in both Experiment 1 (brief duration recognition test) and Experiment 2 (longer duration recall task). Moreover, examination of the drawings from the second experiment showed that patients clearly included the background surfaces in their drawings (e.g., the pebbles behind the bananas) and did so accurately. In fact, although differing in the amount of BE, object details and background content were rated to be indistinguishable for the patients and controls when assessed blindly by a group of independent assessors (see Supplemental Information). Moreover, the patients' remarkable accuracy (mean of 95%) at replacing the borders in the haptic task (a task that permits a large degree of error in terms of area overestimation, see Figure 4A) suggests that the patients attended to and retained the scene details across the testing timeframe. Thus, explanations in terms of differential attention or differential memory for details of the scene do not provide a satisfactory account of our findings. Clearly patients, like controls, recalled scene details and content; what differed was how far beyond the edges of the view they falsely remembered. Patients were less prone to making this error.

Scenes are complex and are composed of multiple elements. In this study, we also attempted to ascertain what aspect of scenes might particularly concern the hippocampus. The results of the scene probe task provided clues in this regard. The patients could accurately describe a given scene, generate a relevant narrative [13], and appropriately characterize the context. When asked to imagine taking a step back from the current position and describe what might then come into view, patients' performance was comparable to that of the control participants; they listed contextually relevant items in the extended scene, associated them with each other, and related them to the context. Thus, the capacity for basic semantic, conceptual, associative, and relational processing was available to the patients. The preservation of these processes, and the intact relationship between objects and backgrounds evident in the patients' performance on the BE tasks, indicates that their reduced BE cannot be easily accommodated by simple relational [39] or binding [40] accounts.

The step-back aspect of the scene probe task also showed that the patients did not have difficulty generating expectancies or predictions about what scene features might be beyond the view, which would have occurred if they could not bring to mind predictable properties associated with the environment. Instead, their self-declared problem was that despite “knowing” what was likely to be there, they could not visualize the space. This was confirmed by an objective analysis of the descriptions of the imagined scene extension, where the patients made virtually no references to space compared with controls, whereas other element types (e.g., people or objects that might be present) were referred to with similar frequency across the two groups.

The patients were impaired at scene construction and in particular their imagined scenes lack spatial coherence. When asked what they thought the problem was, they had some insight; feedback included the following: “There is no scene in front of me here. It's frustrating because I feel like there should be. I feel like I'm listening to the radio instead of watching it on the TV. I'm imagining different things happening, but there's no visual scene opening out in front of me,” “It's as if I have a lot of clothes to hang up in a wardrobe, but there's nothing to hang them on, so they all fall on the floor in a complete mess,” and “It's hard trying to get the space; it keeps getting squashed.” In conjunction with the boundary extension and scene probe findings, this suggests that the legacy of bilateral hippocampal damage may be an inability to internally generate a coherent spatial structure of a scene or environment [41–43]. This accords with the view that for hippocampal-dependent processing, space may be particularly important [2, 3, 6]. We speculate that interference with the ability to internally represent space may prevent the construction of spatially coherent scenes, with possible consequences for navigation, recollection of the past and imagination of the future, which depend on this function. Future work will be needed to investigate this proposed link more directly. Moreover, understanding how the construction of spatially coherent scenes is achieved by the hippocampus, the exact processes and/or representations that are involved, will be critical. BE is an unusual phenomenon that is scene dependent, and it is therefore difficult to conceive of an equivalent temporal test, although consideration should also be given to temporal context in future studies [44].

Boundary extension has received surprisingly little neuroscientific attention. Only one functional magnetic resonance imaging (fMRI) study has examined BE, with a region-of-interest analysis focused on two scene-relevant brain areas, the parahippocampal and retrosplenial cortices [45]. Park et al.'s [45] interest was not in the initial viewing of a scene, the point at which the anticipation of the wider scene occurs, but on retrieval (using an adaptation design), the point at which the BE error is detected. Parahippocampal and retrosplenial cortices responded to scenes and registered the BE error. Given our BE findings and the fact that patients with selective bilateral hippocampal damage cannot construct spatially coherent scenes, we would predict that the hippocampus' involvement is at the initial point of scene extension, although no fMRI study has explicitly examined this as yet. The apparently intact parahippocampal and retrosplenial cortices in our patients, although contributing to scene processing in other ways and perhaps able to compensate for the absent hippocampus to a small degree (in that the patients did not exhibit a complete absence of BE), could not rescue the need to represent surrounding space in a scene, a function we propose is specific to the hippocampus.

## Experimental Procedures

### Participants

Seven patients (three females, mean age 41.43 years, range 32–63 years; mean Full Scale IQ 105, range 99–112) with severe memory impairments associated with bilateral hippocampal damage were tested. Manual segmentation of the individual medial temporal lobe regions (including the hippocampus, parahippocampal, and entorhinal/perirhinal cortices) on high-resolution structural MR images (Figures S1A and S1B), coupled with whole-brain structural MRI and automated voxel-based morphometry (VBM) analysis [46] (Figure S1C), were used to substantiate the selectivity of the hippocampal lesions (see Supplemental Experimental Procedures). Background and details pertaining to each patient are provided in Table S1. All patients were high functioning with no problems in any cognitive domain except memory (see Supplemental Experimental Procedures). In addition, we tested two control participants who exactly matched each patient (two patients, F and G, had similar profiles, and two of the controls matched both of these patients), which gave us a total of 12 age-, sex-, education level-, and IQ-matched control participants (four females, mean age 42.67 years, range 32–63 years; mean Full Scale IQ 109, range 101–115; there were no differences between the groups for age: *U* = 34, Z = −0.68, p = 0.496; or IQ: *U* = 22.5, Z = −1.66, p = 0.097). Only one patient had been tested before in our laboratory (patient A, reported as P04 in [10]). He had no memory of the tasks he performed previously. Each participant gave informed written consent to participation in accordance with the local research ethics committee.

### Data Analysis

Data are presented as mean values ± SD (and graphically in the figures as means ± 1 SEM). For both the behavioral data and manually segmented structural MRI data, statistical significance was calculated by looking at differences in the ranked position order of the scores in the two groups (Mann-Whitney U test). Changes in confidence levels (within subject) across trial categories in the RSVP task were analyzed using the Wilcoxon signed-rank test. Nonparametric statistical analyses were employed due to the small number in our patient group (n = 7). The exceptions to this were one-sample t tests used to assess whether group means on the drawing and haptic tasks differed from a specified constant (i.e., 100%), because no equivalent nonparametric task is available. All tests were two-tailed and differences were considered statistically significant at p < 0.05.

### Additional Tests

The Supplemental Experimental Procedures provides details of a test examining visual illusions (see also Figure S6).

## Supplemental Information

Document S1. Figures S1–S6, Table S1, and Supplemental Experimental Procedures

## References

[1] Andersen P., Morris R., Amaral D., Bliss T., O'Keefe J. (2007). The Hippocampus Book.

[2] Burgess N., Maguire E.A., O'Keefe J. (2002). The human hippocampus and spatial and episodic memory. Neuron.

[3] O'Keefe J., Nadel L. (1978). The Hippocampus as a Cognitive Map.

[4] Scoville W.B., Milner B. (1957). Loss of recent memory after bilateral hippocampal lesions. J. Neurol. Neurosurg. Psychiatry.

[5] Eichenbaum H. (2000). Hippocampus: mapping or memory?. Curr. Biol..

[6] Hassabis D., Maguire E.A. (2007). Deconstructing episodic memory with construction. Trends Cogn. Sci. (Regul. Ed.).

[7] Addis D.R., Wong A.T., Schacter D.L. (2007). Remembering the past and imagining the future: common and distinct neural substrates during event construction and elaboration. Neuropsychologia.

[8] Hassabis D., Kumaran D., Maguire E.A. (2007). Using imagination to understand the neural basis of episodic memory. J. Neurosci..

[9] Szpunar K.K., Watson J.M., McDermott K.B. (2007). Neural substrates of envisioning the future. Proc. Natl. Acad. Sci. USA.

[10] Hassabis D., Kumaran D., Vann S.D., Maguire E.A. (2007). Patients with hippocampal amnesia cannot imagine new experiences. Proc. Natl. Acad. Sci. USA.

[11] Andelman F., Hoofien D., Goldberg I., Aizenstein O., Neufeld M.Y. (2010). Bilateral hippocampal lesion and a selective impairment of the ability for mental time travel. Neurocase.

[12] Klein S.B., Loftus J., Kihlstrom J.J. (2002). Memory and temporal experience: The effects of episodic memory loss on an amnesic patient's ability to remember the past and imagine the future. Soc. Cogn..

[13] Race E., Keane M.M., Verfaellie M. (2011). Medial temporal lobe damage causes deficits in episodic memory and episodic future thinking not attributable to deficits in narrative construction. J. Neurosci..

[14] Rosenbaum R.S., Gilboa A., Levine B., Winocur G., Moscovitch M. (2009). Amnesia as an impairment of detail generation and binding: evidence from personal, fictional, and semantic narratives in K.C. Neuropsychologia.

[15] Intraub H., Richardson M. (1989). Wide-angle memories of close-up scenes. J. Exp. Psychol. Learn. Mem. Cogn..

[16] Gottesman C.V., Intraub H. (2002). Surface construal and the mental representation of scenes. J. Exp. Psychol. Hum. Percept. Perform..

[17] Intraub H., Gottesman C.V., Bills A.J. (1998). Effects of perceiving and imagining scenes on memory for pictures. J. Exp. Psychol. Learn. Mem. Cogn..

[18] Seamon J.G., Schlegel S.E., Hiester P.M., Landau S.M., Blumenthal B.F. (2002). Misremembering pictured objects: people of all ages demonstrate the boundary extension illusion. Am. J. Psychol..

[19] Candel I., Merckelbach H., Houben K., Vandyck I. (2004). How children remember neutral and emotional pictures: boundary extension in children's scene memories. Am. J. Psychol..

[20] Quinn P.C., Intraub H. (2007). Perceiving “outside the box” occurs early in development: evidence for boundary extension in three- to seven-month-old infants. Child Dev..

[21] Intraub H. (2012). Rethinking visual scene perception. Wiley Interdisciplinary Reviews: Cognitive Science.

[22] Dickinson C.A., Intraub H. (2008). Transsaccadic representation of layout: what is the time course of boundary extension?. J. Exp. Psychol. Hum. Percept. Perform..

[23] Intraub H., Dickinson C.A. (2008). False memory 1/20th of a second later: what the early onset of boundary extension reveals about perception. Psychol. Sci..

[24] Intraub H., Gottesman C.V., Willey E.V., Zuk I.J. (1996). Boundary Extension for briefly glimpsed photographs: Do common perceptual processes result in unexpected memory distortions?. J. Mem. Lang..

[25] Gottesman C.V., Intraub H. (1999). Wide-angle memories of close-up scenes: a demonstration of boundary extension. Behav. Res. Methods Instrum. Comput..

[26] Intraub H. (2004). Anticipatory spatial representation of 3D regions explored by sighted observers and a deaf-and-blind-observer. Cognition.

[27] Hubbard T.L., Hutchison J.L., Courtney J.R. (2010). Boundary extension: findings and theories. Q J Exp Psychol (Hove).

[28] Graham K.S., Barense M.D., Lee A.C. (2010). Going beyond LTM in the MTL: a synthesis of neuropsychological and neuroimaging findings on the role of the medial temporal lobe in memory and perception. Neuropsychologia.

[29] Roediger H.L.I. (1996). Memory Illusions. J. Mem. Lang..

[30] Eichenbaum H., Fagan A., Mathews P., Cohen N.J. (1988). Hippocampal system dysfunction and odor discrimination learning in rats: impairment or facilitation depending on representational demands. Behav. Neurosci..

[31] Poldrack R.A., Packard M.G. (2003). Competition among multiple memory systems: converging evidence from animal and human brain studies. Neuropsychologia.

[32] Saksida L.M., Bussey T.J., Buckmaster C.A., Murray E.A. (2007). Impairment and facilitation of transverse patterning after lesions of the perirhinal cortex and hippocampus, respectively. Cereb. Cortex.

[33] Schacter D.L. (1996). Illusory memories: a cognitive neuroscience analysis. Proc. Natl. Acad. Sci. USA.

[34] Verfaellie M., Schacter D.L., Cook S.P. (2002). The effect of retrieval instructions on false recognition: exploring the nature of the gist memory impairment in amnesia. Neuropsychologia.

[35] Eichenbaum H., Kapur N. (2011). The paradoxical hippocampus: When forgetting helps learning. The Paradoxical Brain.

[36] Kapur N., Kapur N. (2011). Paradoxical functional facilitation and recovery in neurological and psychiatric conditions. The Paradoxical Brain.

[37] Intraub H., Bodamer J.L. (1993). Boundary extension: fundamental aspect of pictorial representation or encoding artifact?. J. Exp. Psychol. Learn. Mem. Cogn..

[38] Intraub H., Daniels K.K., Horowitz T.S., Wolfe J.M. (2008). Looking at scenes while searching for numbers: dividing attention multiplies space. Percept. Psychophys..

[39] Cohen N.J., Eichenbaum H. (1993). Memory, Amnesia, and the Hippocampal System.

[40] Ranganath C. (2010). A unified framework for the functional organization of the medial temporal lobes and the phenomenology of episodic memory. Hippocampus.

[41] Komorowski R.W., Manns J.R., Eichenbaum H. (2009). Robust conjunctive item-place coding by hippocampal neurons parallels learning what happens where. J. Neurosci..

[42] Manns J.R., Eichenbaum H. (2009). A cognitive map for object memory in the hippocampus. Learn. Mem..

[43] Moita M.A., Rosis S., Zhou Y., LeDoux J.E., Blair H.T. (2003). Hippocampal place cells acquire location-specific responses to the conditioned stimulus during auditory fear conditioning. Neuron.

[44] MacDonald C.J., Lepage K.Q., Eden U.T., Eichenbaum H. (2011). Hippocampal “time cells” bridge the gap in memory for discontiguous events. Neuron.

[45] Park S., Intraub H., Yi D.J., Widders D., Chun M.M. (2007). Beyond the edges of a view: boundary extension in human scene-selective visual cortex. Neuron.

[46] Ashburner J., Friston K.J. (2005). Unified segmentation. Neuroimage.

