# Supplementary material for: Attenuated Boundary Extension Produces a Paradoxical Memory Advantage in Amnesic Patients
Source: Curr Biol. 2012 Feb 21;22(4):261–8. doi: 10.1016/j.cub.2012.01.001 (PMC3315012; doi:10.1016/j.cub.2012.01.001)
Supplement: Document S1. Figures S1–S6, Table S1, and Supplemental Experimental Procedures [file mmc1.pdf]

Current Biology, Volume *22*

Supplemental Information

Attenuated Boundary Extension Produces a Paradoxical Memory Advantage in Amnesic Patients

Sinéad L. Mullally, Helene Intraub, and Eleanor A. Maguire

Supplemental Inventory

1. Supplemental Figures and Tables

Table S1

Figure S1

Figure S2

Figure S3, related to Figure 2

Figure S4, related to Figure 3

Figure S5, related to Figure 5

Figure S6

2. Supplemental Experimental Procedures

3. Supplemental References

Table S1. Summary of Patient Details

ID	Sex	Age (yrs)	H'ness	Education Level	Aetiology	Chronicity (yrs)	Full Scale IQ	Matrix[a] Reasoning	Retrograde Amnesia (yrs)	Complex Figure[b]	Recognition[c]	Recall[c]
A	M	63	R	University	LE	6	99	11	10	10	Unimpaired	Impaired[d]
B	M	40	R	A-Levels	Anoxia	21	112	16	<1	2	Unimpaired	Impaired
C	M	37	R	University	LE	3.5	107	11	~25	1	Impaired	Impaired
D	F	32	R	University	LE	7	109	13	10	4	Borderline	Impaired
E	M	38	R	GCSE	LE	3.5	99	8	1	1	Impaired	Impaired
F	F	40	R	University	Unknown	22	105	14	18	5	Unimpaired	Impaired[d]
G	F	40	L	University	Unknown	24	105	13	16	5	Unimpaired	Impaired[d]

H'ness = handedness; Chronicity = number of years since the illness/incident precipitating the hippocampal damage/memory loss; A-Levels = school examinations taken at the point of leaving secondary school ~18 years of age; GCSE = school examinations taken ~14-16 years of age; LE = limbic encephalitis. [a]Scaled score, matrix reasoning subtest of the WAIS-III; [b]Complex Figure (Rey-Osterrieth /BMIBP) delayed recall (percentile score); [c]see text for further details; [d]impaired after an extended delay.

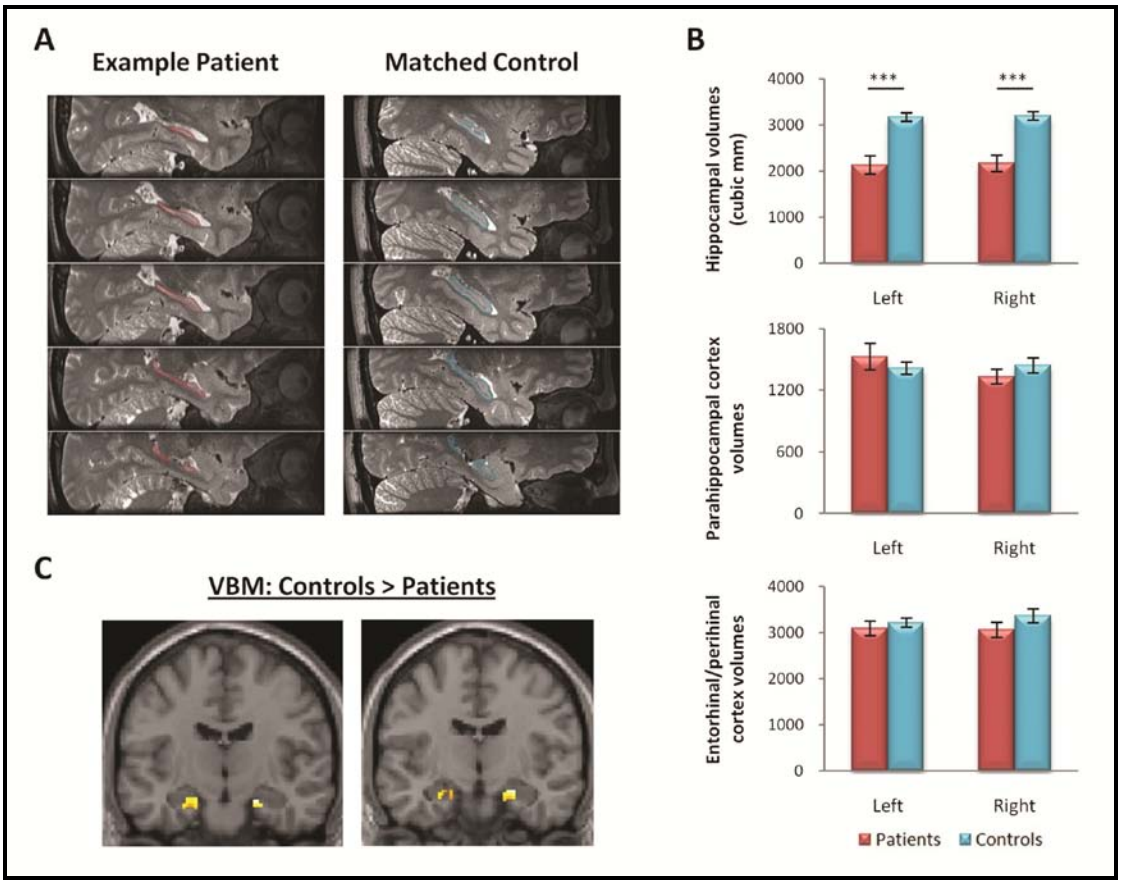

Figure S1. Lesion Characterisation

(A) An example high-resolution T2-weighted scan from a hippocampal patient and one of their matched controls. The outer boundary of the hippocampus is illustrated in red for the patient and blue for the control.

(B) Volumetric measurements were extracted from the manually segmented medial temporal lobe regions. Significant reductions in hippocampal volume were observed in the patient group relative to the matched control group (***$P < 0.001$). By contrast, no significant volume differences were found in the parahippocampal or entorhinal/perirhinal cortices.

(C) An automated VBM analysis was performed to identify regions, at a whole-brain level, where grey matter volume differed significantly between the control and patient groups. Selective reductions in grey matter volume were observed in the patients, relative to the control group, only in the left and right hippocampus (images are displayed at $P < 0.05$ uncorrected).

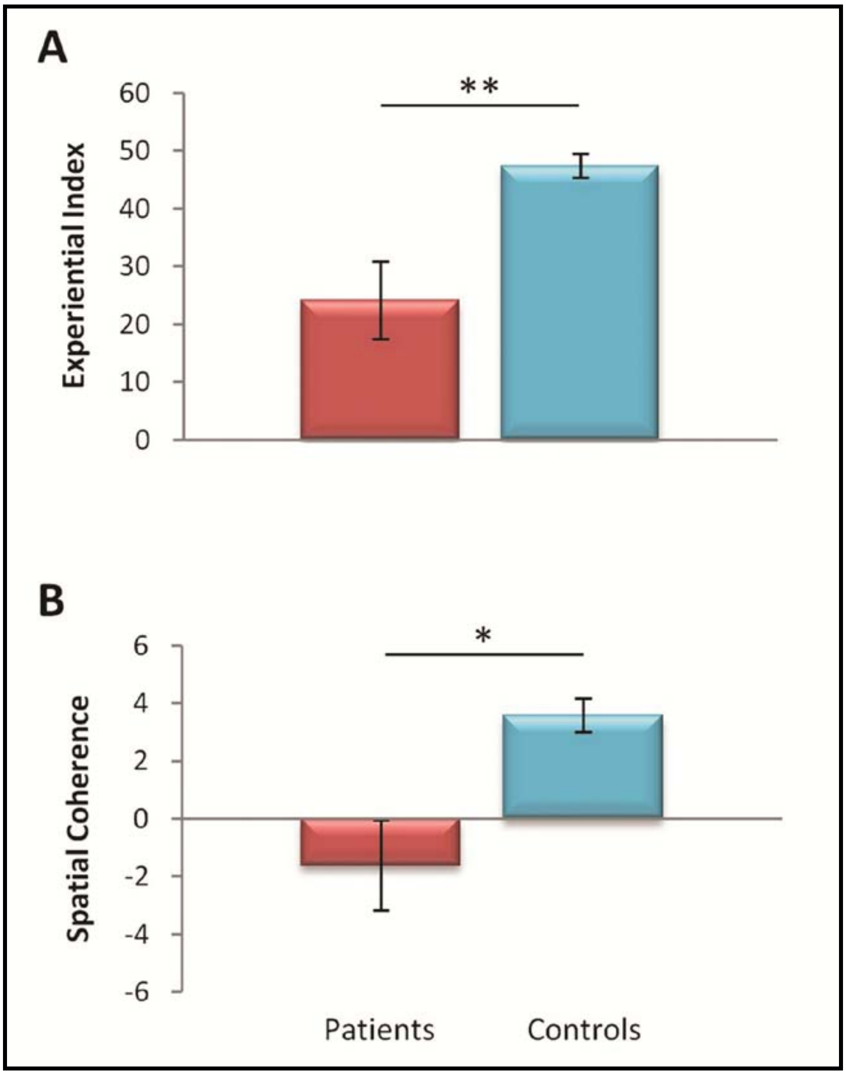

Figure S2. Scene Construction Task

In this abbreviated three-trial version of the scene construction task, the cues related to scenes in a swimming pool, a harbour and a library. Administration and scoring procedures were identical to those used in the previously published protocol [1]. An overall measure of the richness of the imagined experiences, the experiential index (EI), was derived (0, not experienced at all…60, extremely richly experienced), as was a measure of the contiguousness and spatial integrity of the imagined scenes using the spatial coherence index (-6, fragmented ...+6, spatially coherent). Relative to the matched control participants, the patients with selective bilateral hippocampal damage and amnesia constructed (A) impoverished (experiential index) and (B) less spatially-coherent (spatial coherence index) fictitious scenes (*$P < 0.05$; ** $P < 0.01$).

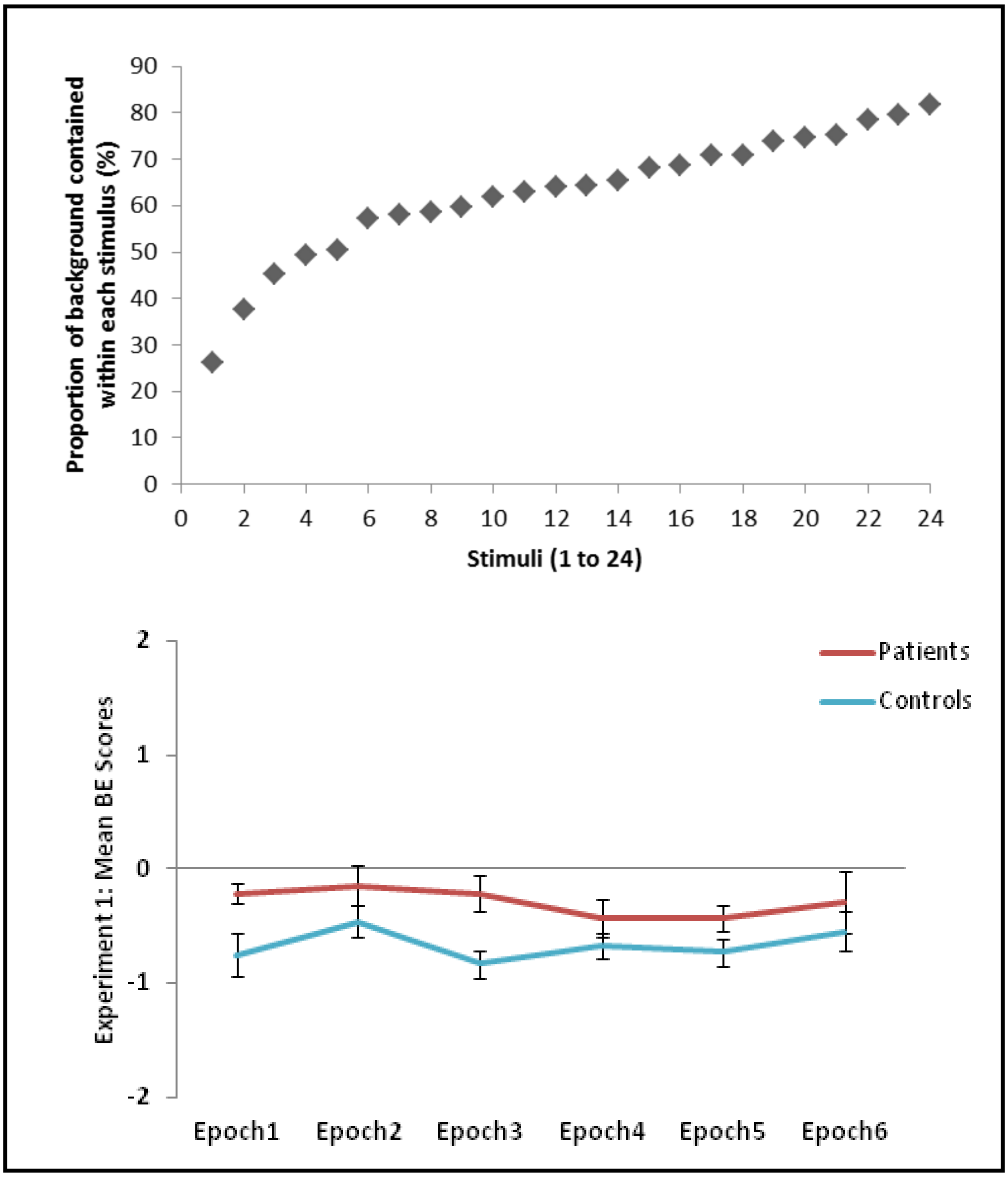

Figure S3. Analysis of Stimuli in Experiment 1, the RSVP Task

The upper panel shows the proportion of background within each of the twenty-four stimuli (note the stimuli were presented in a random order and not that shown). The variation is clearly evident, allowing us to be confident that there were no regularities that could be learned in this stimulus set. The lower panel shows the 24 trials divided into six epochs (four trials/epoch). The data are represented in terms of the mean boundary extension scores. These are calculated by averaging the ratings (much closer-up = -2; 'a little closer-up' = -1; 'the same' = 0; 'a little farther away' = +1; 'much farther away' = +2) made by each participant within each epoch. Thus, a mean score of '0' reflects no BE, while a minus score reflects BE. We found no evidence that the patients showed less BE as the task progressed, showing that a learning effect cannot account for our findings. Moreover, the between-group difference was clearly evident within the first four trials (epoch 1) where a learned regularity would likely not yet have been established.

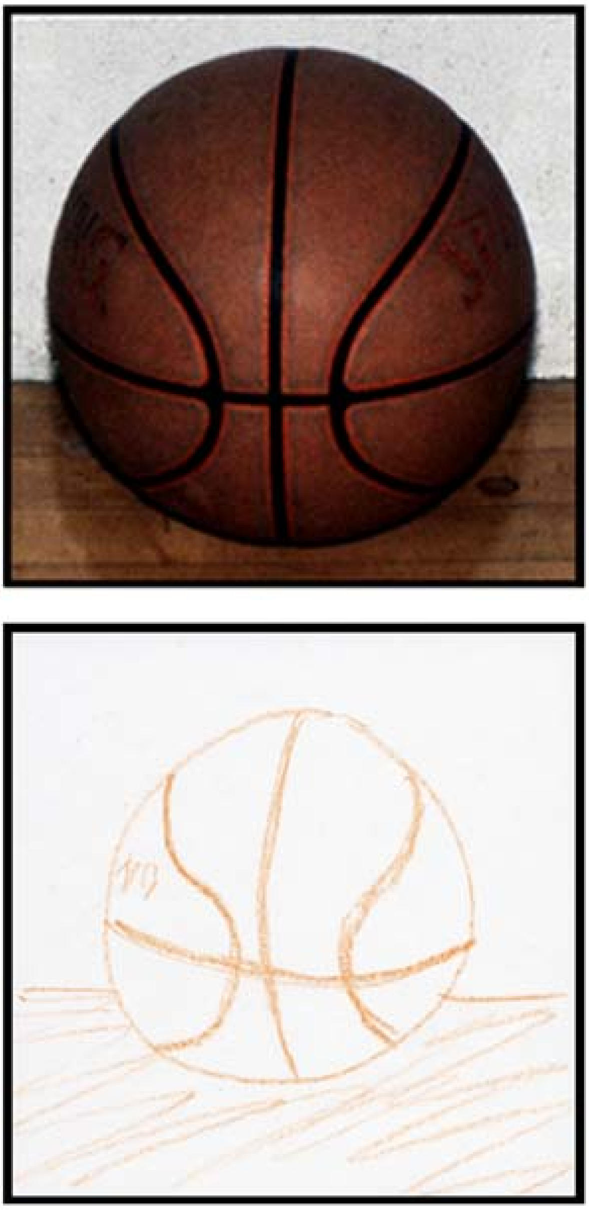

Figure S4. Example Trial from the Drawing Raters' Tasks

The top panel shows the original stimulus. The lower panel shows a participant's drawing. See Supplemental Experimental Procedures for details of the tasks the assessors undertook.

Q1) Can you describe the object in this picture?
"It's a park bench".

Q2) Can you describe the background in this picture?
"Yeah, looks like a factory building. There's a lead roof, brick building, 4 windows, a chimney pot. The buildings maybe 80 to 100 years old. Definitely an old building. There is a low window to the left of the v-shaped tree. Sky in the background. There are bushes; long thin branches coming from the ground".

Q3) Can you describe the colours in this picture?
"Soil is brown. Grass is green. Browns and greens mostly".

Q4) What sort of a place do you think this picture was taken in?
"Either a cemetery or church grounds".

Q5) If you were taking the picture and you took a few steps backwards, what else do you think would come into view?

"Birds[EP] possible. [More leaves]. Another bench[EP]. Old[SD] ladies[EP]. Possibly [old] men[EP]. And small, very small[SD] children[EP], with grandparents[EP]. And animals, dogs[EP]... making a mess of all the grounds[TEA]. Possibly plastic[SD] bags[EP]. The ladies would be tidying up after the dogs[TEA]. Possibly also bins[EP], connected to[TEA] lamp posts[EP]... [so lamp posts]. A path[EP]. A fence[EP]. A gate[EP]. A church[EP]. A headstone[EP]".

Q6) 'Can you visualise the scene with these extra details or do you just know that this is what else is likely to be there?
"I just know what is likely to be there."

Figure S5. Example Responses from a Patient on the Scene Probe Task

This patient, like all the other patients, responded appropriately and effortlessly to the first four questions. However, the absence of spatial references in the patient's response to question 5 is evident in this extract. Moreover, his inability to truly visualise the extended scene in his mind's eye is clearly apparent in his response to the final question. Taken together, this patient's responses to questions 5 and 6 illustrate the striking juxtaposition between the patients' deficits in imagining spatial frameworks and their preserved ability to bring to mind appropriate semantic and contextual scene associations.

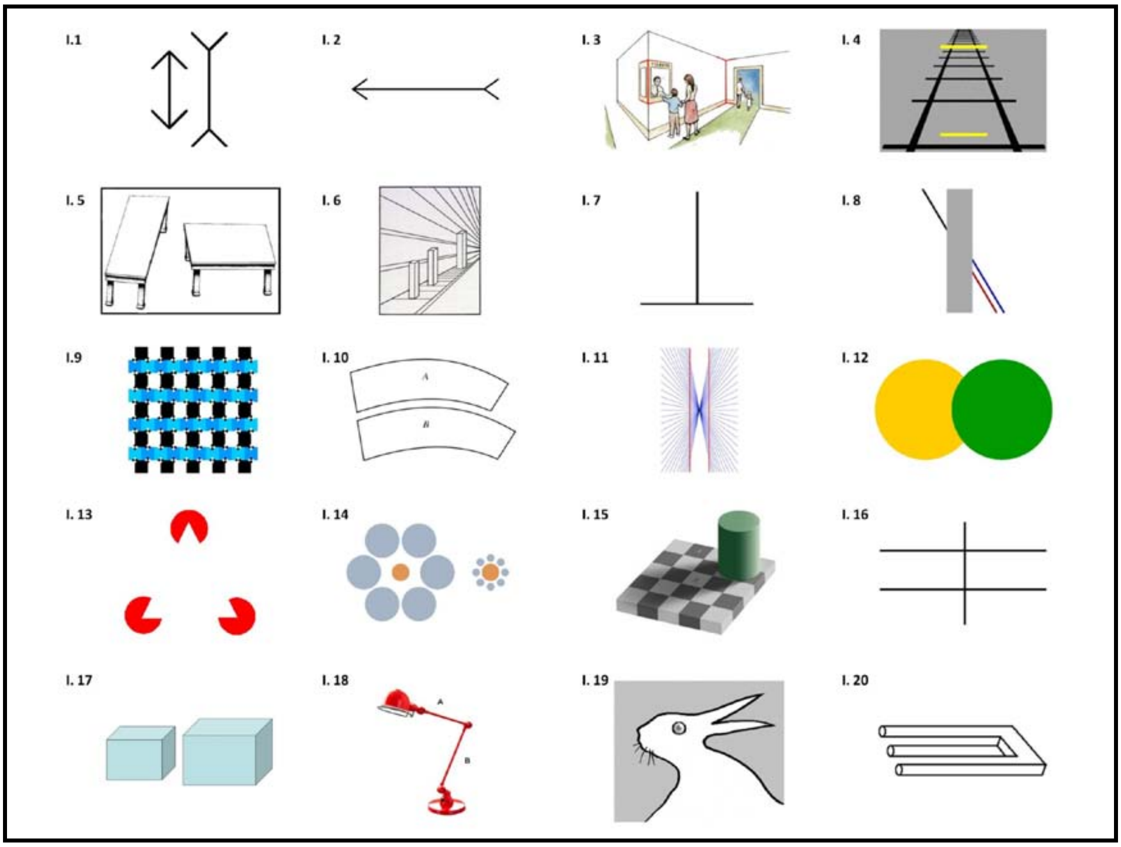

Figure S6. The Visual Illusions Task

The set of common visual illusions (plus lures) used to probe participants' responses to perceptual distortions.

Supplemental Experimental Procedures

Patient Details

Background details of the patients are provided on Table S1. Of the seven patients, five had either suffered anoxia (patient B: carbon monoxide poisoning aged 19), or limbic encephalitis (patients A, C-E). The two other patients (patients F and G) presented with long-standing, significant memory and navigation problems, and a profound inability to visualise or imagine anything. In neither case was a specific illness or incident identifiable, although both had selective and significantly reduced hippocampal volumes relative to matched controls (patient F: left hippocampus (LHC) reduced to 78.33%, right hippocampus (RHC) 72.16%; patient G: LHC 68.70%, RHC 73.31%; see more below on lesion assessment). In the case of patient F, the problems became apparent around the age of 18, and for patient G slightly earlier around the age of 16. Both patients report that they have no memories of specific episodes and events that had happened to them before (or since) the time their problems emerged, although they have semantic and general knowledge about their lives. Both patients rely heavily on detailed diaries and other memory aids to assist them through life. Their determination and efforts meant that both were able to attain a university education (computer science/social work), but admitted it had been a struggle.

Neuropsychology

As is evident on Table S1, all patients had IQs in the average range or above, with scaled scores average or above on block design, and attention (digit span). In addition, patients scored generally above average on the matrix reasoning test, demonstrating excellent visuo-perceptual and reasoning skills; they also had near perfect scores on the dot location and cube analysis subtests of the Visual and Object Space Perception (VOSP) Battery, and perfect copying of a complex figure. Patients scored average or better on the Brixton Spatial Anticipation Test, a measure of frontal-executive functioning, and also in the average range or above on tests of verbal fluency (patient A could not be tested on the latter as he was not a native English speaker). Overall, the patients were high functioning, with no problems in any cognitive domain except memory.

Retrograde memory was assessed using in-depth clinical interviews with the patients and a family member (typically a spouse). The extent of retrograde amnesia (RA) is shown on Table S1, and varied from less than 1 year to a lifetime pre-lesion, with a mean RA of 12.14 years. Of note, the very supportive families of the patients worked hard to remind them of events in their past, using photographs and discussions. In particular the RA of patients B and E may be underestimates as the patients and their families acknowledged that their interventions made it difficult to ascertain the true extent of pre-morbid memory loss.

Anterograde memory was assessed using standard clinical measures of recognition (Warrington Recognition Memory Test for Faces and Words/Recognition Test for the story recall from the Wechsler Memory Scale III), and recall (immediate and delayed story recall from the Wechsler Memory Scale III, or the stories from the BMIPB – a British-normed test similar to the Wechsler Memory Scale; delayed recall of the Rey-Osterrieth Complex Figure, or the complex figure from the BMIPB). There were no obvious laterality differences, with each patient performing similarly for visual and verbal material. In terms of recognition, performance across patients varied from impaired to borderline to unimpaired. There was no relationship with extent of hippocampal volume loss. This echoes the inconsistencies in the amnesia literature with regard to recognition memory in the context of hippocampal amnesia. By contrast, all patients had significant deficits on the recall tests, with patients B, C, D, and E almost at floor on all tests. Patients A, F and G faired slightly better performing below average at the standard 30 minutes delayed recall, but thereafter experienced rapid

forgetting. There was no difference between these three patients and the other four in terms of their performance on the boundary extension and other experimental tests.

Lesion Characterisation

High-resolution structural MR images were acquired in a partial volume focused on the temporal lobes (Figure S1A) using a 3T whole body MRI scanner (Magnetom TIM Trio, Siemens Medical Solutions, Erlangen, Germany). This was operated with the standard transmit body coil and 32-channel head receive coil. Full details of the sequence have been described elsewhere [2]. To summarise, a single-slab 3D T2-weighted turbo spin echo sequence with variable flip angles, in combination with parallel imaging was employed to simultaneously achieve a high image resolution of ~500 µm, high sampling efficiency and short scan time while maintaining a sufficient signal-to-noise ratio (SNR). After excitation of a single axial slab the image was read out with the following parameters: resolution = 0.52 x 0.52 x 0.5 mm^3, matrix = 384 x 328, partitions = 104, partition thickness = 0.5 mm, partition oversampling = 15.4%, field of view = 200 x 171 mm2, TE = 353 ms, TR = 3200 ms, GRAPPA x 2 in phase-encoding (PE) direction, bandwidth = 434 Hz/pixel, echo spacing = 4.98 ms, turbo factor in PE direction = 177, echo train duration = 881, averages = 1.9. For reduction of signal bias due to, e.g. spatial variation in coil sensitivity profiles, the images were normalized using a prescan and a weak intensity filter was applied as implemented by the scanner's manufacturer. To improve the SNR of the anatomical image, four scans were acquired for each participant, co-registered and averaged.

The hippocampus, posterior parahippocampal cortex, and entorhinal/perirhinal cortices were manually segmented using ITK-SNAP32 (version 2.0, www.itksnap.org) [3] and in accordance with established anatomical guidelines [4, 5]. Volumetric measurements (in cubic mm) were extracted for each region[1]. Significant volume reductions were evident in the patient group, relative to the control group, in both the left (U = 2, Z = -3.38, P < 0.001) and the right (U = 2, Z = -3.38, P < 0.001) hippocampus (Figure S1B). This corresponded to a mean volume reduction of 32%. No significant between-group differences were found in the posterior parahippocampal cortex (left: U=38, Z = -0.34, P = 0.74; right: U = 33, Z = -0.76, P = 0.45) or in the entorhinal/perirhinal cortices (left: U = 32, Z = -0.85, P = 0.40; right: U = 25, Z = -1.44, P = 0.15). Results were highly similar with or without correction for intra-cranial volume (the latter are reported here). Inter-rater reliability was assessed in a random sample of the segmentations. A high degree of overlap was observed across all segmented regions from the two raters, one of whom was blind to participant status (DICE scores: hippocampus: 0.85; parahippocampal cortex 0.81; entorhinal/perirhinal cortex 0.80).

Whole-brain structural MR T1-weighted images (1mm^3 voxels) were also acquired on all participants using an optimised 3D Modified Driven Equilibrium Fourier Transform (MDEFT) sequence [6] on the same 3T Trio scanner (32-channel head receive coil). All images were visually inspected for structural damage beyond the hippocampus and medial temporal

---

[1] In two patients for whom high-resolution structural MR images could not be acquired, the T1-weighted whole brain MRI scans were used for manual segmentation using the same anatomical guidelines as for the high resolution scans. In order to ensure that volumes extracted from the high-resolution T2-weighted partial volume scans and the T1-weighted whole-brain scans were comparable, we repeated the manual segmentation on these patients' matched controls for whom we had acquired both the high-resolution T2-weighted partial volume scans and the T1-weighted whole-brain scans. No notable differences were observed in the volumes extracted from either scan type in any of the segmented regions. The volumes extracted from the T1 whole-brain scans for these two patients and their matched controls were therefore entered into the overall volumetric analysis.

lobes (MTL). No additional damage was observed in any of the patients. An automated voxel-based morphometry (VBM) analysis [7] was then performed on these whole-brain images to look for consistent changes in grey matter volume anywhere in the brain. It was implemented in SPM8 (www.fil.ion.ucl.ac.uk/spm) using a smoothing kernel of 8mm. Given the small size of our samples, significance level was set at $P < 0.01$ uncorrected for multiple comparisons (Figure S1C). In agreement with the data acquired from the manual segmentations, decreases in grey matter volume were evident only in the left (-20, -16, -20, Z = 2.43) and right (22, -12, -20, Z = 2.46; 24, -10, -18, Z = 2.38) hippocampi.

Additional Ratings of Drawings from the BE Drawing Task

Five raters, who were naïve about BE and the participants in our study, were shown the drawings of the patients and control participants one at a time on a computer screen (see Figure S4). Each drawing was accompanied by the relevant original stimulus. Drawing presentation was randomised across participant group (patients, controls) and scene type (basketball, bananas, bucket and spade). The rater was required to decide on each trial if the drawing was made by a healthy control participant or a person with a memory problem. They were told to consider how accurately the objects and the backgrounds had been depicted (irrespective of general drawing ability). They had up to 10 seconds to respond on each trial, although they typically responded more quickly. Having made their decisions, they were asked on what basis they had done so. All raters stated that they paid particular attention to the object details (e.g. the lines on the basketball) as well as the overall content of the backgrounds. Across the raters, 56.33% (SD 27.37) of patients were misclassified as controls, and 37.78% (SD 25.38) of controls were misclassified as patients. When these ratings were compared to chance (i.e. to 50%), neither the patient ($t = 0.52$, $df = 4$, $P > 0.05$) nor control ($t = -1.08$, $df = 4$, $P > 0.05$) drawings were classified by raters any better than chance. This shows that the patients and controls could not be clearly differentiated in terms of the object details or the overall background content in their drawings.

In a second study, a new set of five assessors blindly rated the different elements of the drawings (i.e. the objects and the backgrounds) separately (see Figure S4). First they had to evaluate each drawing to answer this question: “Regardless of artistic ability, and ignoring the background, does the OBJECT in the drawing below capture what is depicted in the picture?” They selected from among the following options: Not at all (1), A little (2), A lot (3), Exactly (4). There was no significant difference in ratings for the objects for patients [mean 2.80 (0.30)] and controls [2.89 (0.37); $P > 0.05$]. The assessors then had to answer the following question: “Regardless of artistic ability, and ignoring the object, does the BACKGROUND in the drawing below capture what is depicted in the picture?”, with same response options as for the object question. There was no significant difference in ratings for the backgrounds for patients [mean 2.30 (0.38)] and controls [2.42 (0.37); $P > 0.05$].

Visual Illusions Task

Although the BE effect is revealed once the original stimuli have been removed, the superficial similarity of BE to a visual illusion prompted us to examine how the patients would respond to well-known visual illusions (where, unlike our BE tasks, the stimuli remain in view for the duration of a trial). The task consisted of sixteen well-known visual illusions which were briefly presented to participants (Figure S6). Participants were asked a simple question after each trial. The question was designed in such a way as to avoid biasing participants’ responses. Examples of questions included: Illusion 1: ‘Is one of the lines longer than the other, or are they the same?’; Illusion 5: ‘Are these table tops the same or different?’; Illusion 2: ‘Mark where you think the centre of the line is’; Illusion 20: ‘Draw the picture that you just saw’. Four ‘lures’ (non-illusions) were also included (e.g. Illusion 12: ‘Is

one of these circles bigger than the other, or are they both the same? See also lures 16, 17 and 18). This task was completed by a subset of the patients (n=5) and controls (n=9), and the frequency of the expected responses was compared between the two groups (using chi-squared analyses). As anticipated, patients performed normally on all of the illusions (i.e. they, like controls, were tricked by the illusions), including those that required an interpretation of spatial properties such as depth. In addition, both groups performed highly accurately on the lures. Therefore, while the patients cannot internally generate coherent spatial frameworks (i.e. they were unable to construct scenes, and had reduced BE), their basic processing of visuo-spatial information was unimpaired (see also [8]).

Supplemental References

1. Hassabis, D., Kumaran, D., Vann, S.D., and Maguire, E.A. (2007). Patients with hippocampal amnesia cannot imagine new experiences. Proc Natl Acad Sci U S A *104*, 1726-1731.
2. Bonnici, H.M., Kumaran, D., Chadwick, M.J., Weiskopf, N., Hassabis, D., and Maguire, E.A. (2011). Decoding representations of scenes in the medial temporal lobes. Hippocampus.
3. Yushkevich, P.A., Piven, J., Hazlett, H.C., Smith, R.G., Ho, S., Gee, J.C., and Gerig, G. (2006). User-guided 3D active contour segmentation of anatomical structures: significantly improved efficiency and reliability. Neuroimage *31*, 1116-1128.
4. Duvernoy, H.M. (2005). The Human Hippocampus, (Berlin: Springer Verlag).
5. Insausti, R., Juottonen, K., Soininen, H., Insausti, A.M., Partanen, K., Vainio, P., Laakso, M.P., and Pitkanen, A. (1998). MR volumetric analysis of the human entorhinal, perirhinal, and temporopolar cortices. AJNR Am J Neuroradiol *19*, 659-671.
6. Deichmann, R., Schwarzbauer, C., and Turner, R. (2004). Optimisation of the 3D MDEFT sequence for anatomical brain imaging: technical implications at 1.5 and 3 T. Neuroimage *21*, 757-767.
7. Ashburner, J., and Friston, K.J. (2005). Unified segmentation. Neuroimage *26*, 839-851.
8. Lee, A.C., and Rudebeck, S.R. (2010). Human medial temporal lobe damage can disrupt the perception of single objects. J Neurosci *30*, 6588-6594.
